# Supplementary figures and images for: RatLesNetv2: A Fully Convolutional Network for Rodent Brain Lesion Segmentation
Source: Front Neurosci. 2020 Dec 22;14:610239. doi: 10.3389/fnins.2020.610239 (PMC7783408; doi:10.3389/fnins.2020.610239)

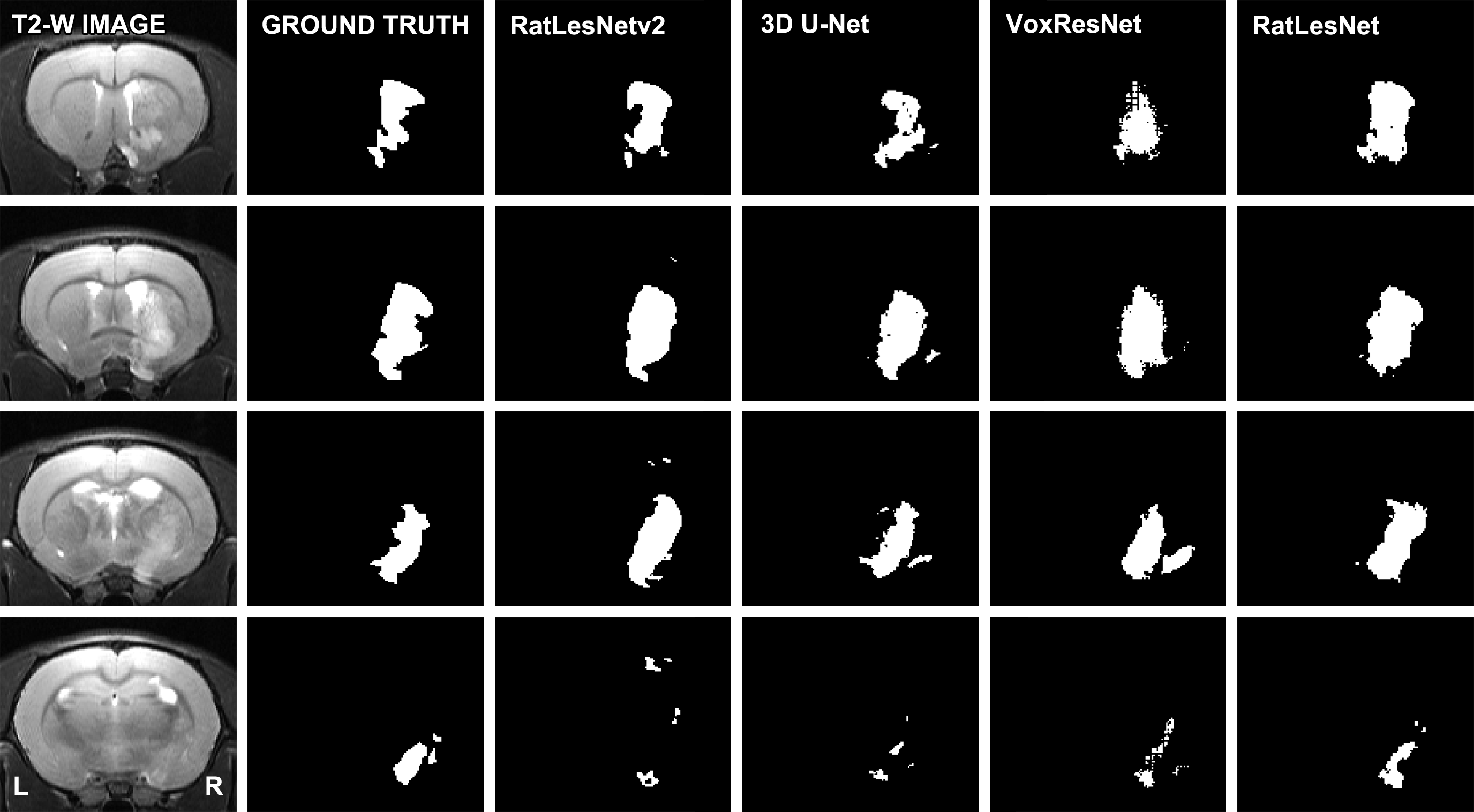

Supplement: Supplementary file 2 [file Image_1.PNG]

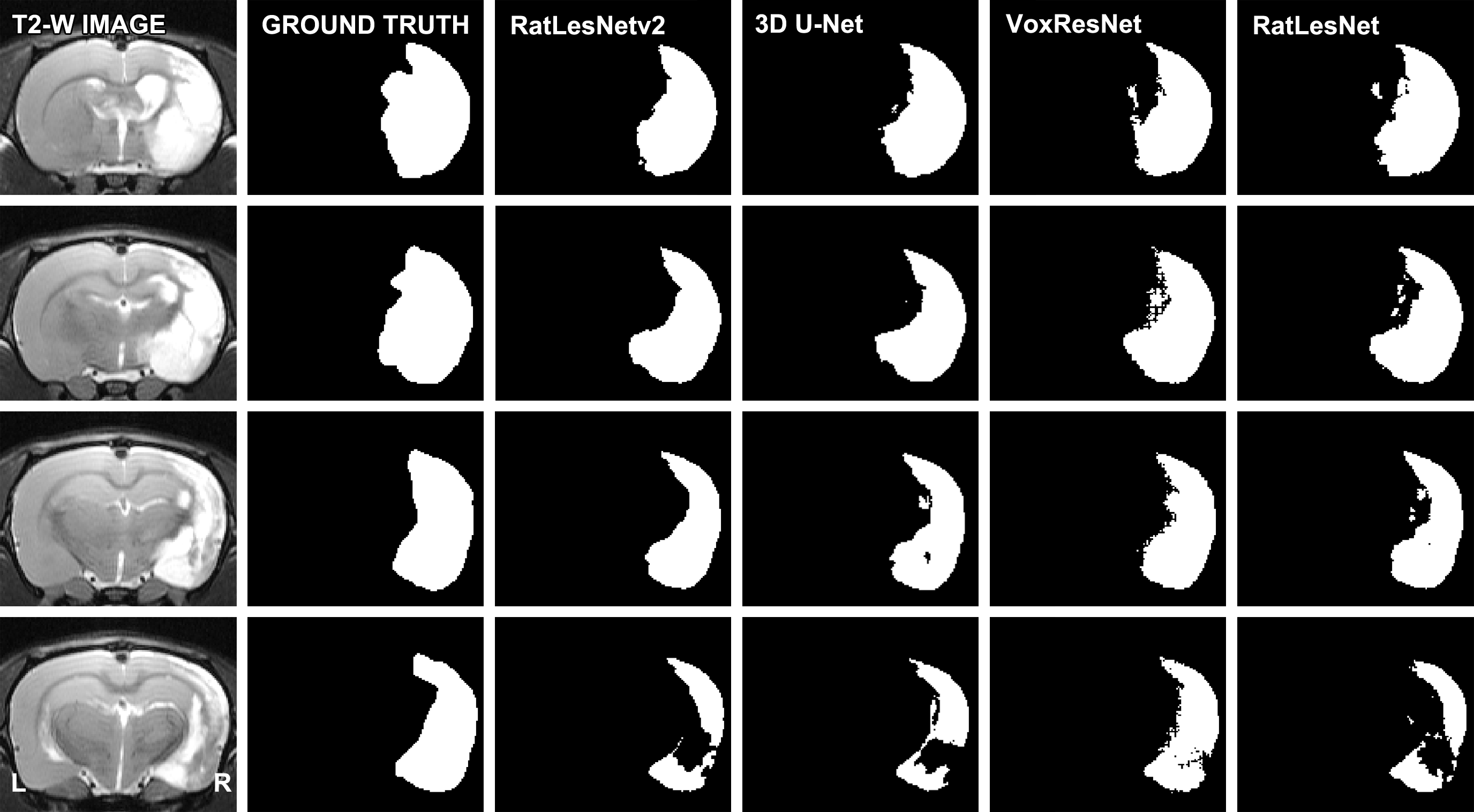

Supplement: Supplementary file 3 [file Image_2.PNG]

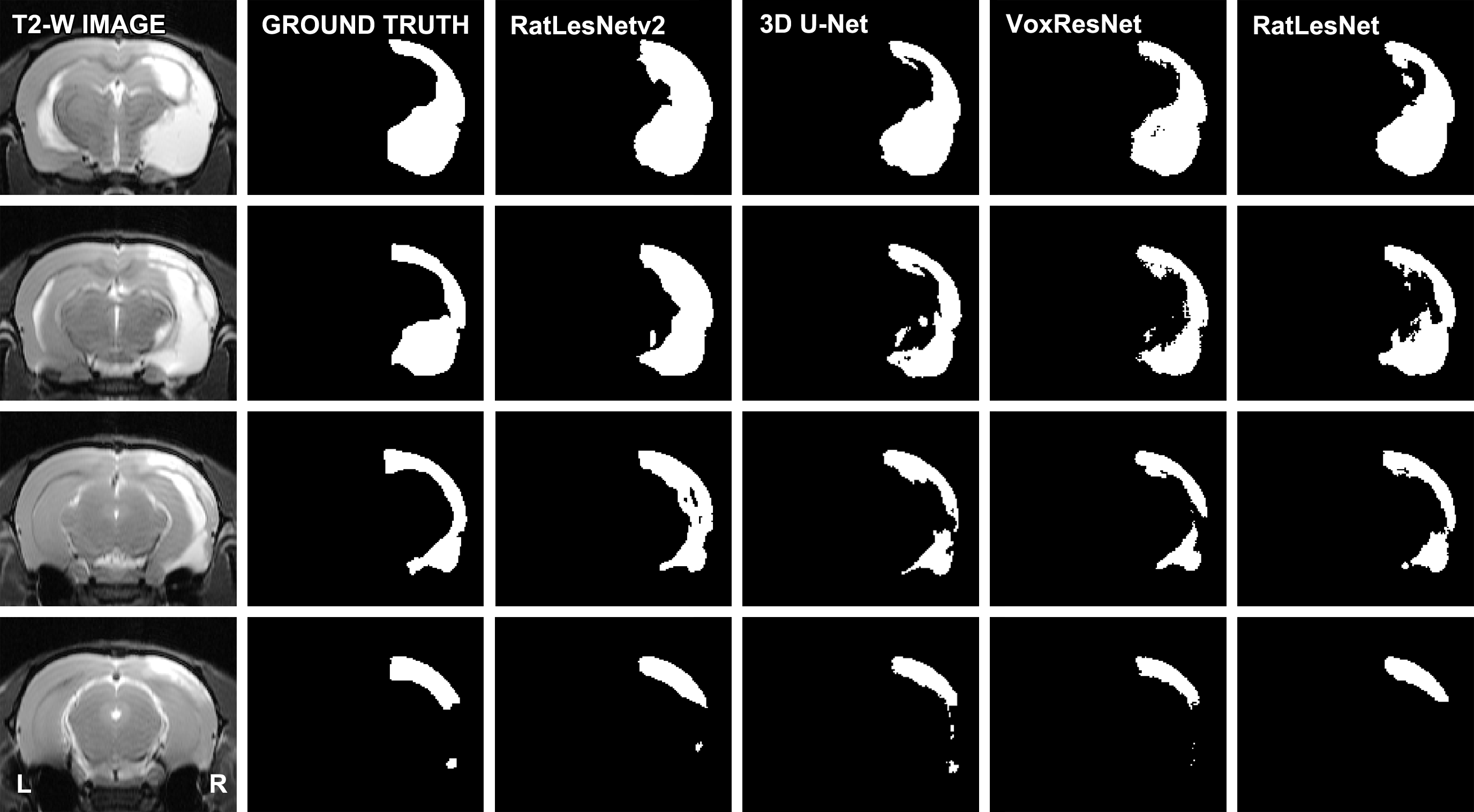

Supplement: Supplementary file 4 [file Image_3.PNG]
